# Providing informal care in a changing society

**DOI:** 10.1007/s10433-016-0370-7

**Published:** 2016-04-15

**Authors:** Marjolein I. Broese van Groenou, Alice De Boer

**Affiliations:** 1 VU University, Amsterdam, The Netherlands; 2 The National Institute for Social Research, The Hague, The Netherlands

**Keywords:** Informal care, Ageing, Theory, Society

## Abstract

The ageing of society is leading to significant reforms in long-term care policy and systems in many European countries. The cutbacks in professional care are increasing demand for informal care considerably, from both kin and non-kin. At the same time, demographic and societal developments such as changing family structures and later retirement may limit the supply of informal care. This raises the question as to whether the volume of informal care (in people) will increase in the years ahead. This paper aims to provide a theoretical answer to this question in two steps. First, based on different care models and empirical literature, we develop a behavioural model on individual caregiving, the Informal Care Model. The model states that, in response to the care recipient’s need for care, the intention to provide care is based on general attitudes, quality of the relationship, normative beliefs, and perceived barriers. Whether one actually provides care also depends on the care potential of the social context, being the family, the social network, and the community. Second, we discuss how current policy and societal developments may negatively or positively impact on these mechanisms underlying the provision of informal care. Given the increased need for care among home-dwelling individuals, the model suggests that more people will take up the caregiver role in the years ahead contributing to larger and more diverse care networks. It is concluded that long-term informal care provision is a complex phenomenon including multiple actors in various contexts. More research is needed to test the Informal Care Model empirically, preferably using information on care recipients, informal caregivers and community care in a dynamic design and in different countries. Such information will increase insight in the developments in informal care provision in retrenching welfare states.

## Introduction

Population ageing is increasing the need for alternatives to publicly provided long-term care in European societies. Many Western European countries are currently implementing major reforms of long-term care, generally accompanied by a vibrant discourse on civic responsibility and civic values with regard to self-care and helping others (Pavolini and Ranci 2008). Governments are pinning increased reliance on informal caregivers to compensate for the cutbacks in residential and professional home care. Informal care is generally defined as the unpaid care provided to older and dependent persons by a person with whom they have a social relationship, such as a spouse, parent, child, other relative, neighbour, friend or other non-kin (Triantafillou et al. 2010). This may involve help with household chores or other practical errands, transport to doctors or social visits, social companionship, emotional guidance or help with arranging professional care. The volume of informal care is already relatively large. In most European countries, the majority of the care received by those aged over 50 is informal care (Verbeek-Oudijk et al. 2014), and about a third of the over-50s provide help with instrumental tasks and/or personal care to an older dependent person (Colombo et al. 2011: 88). On average, one-third of informal caregivers in OECD countries provide care to their spouse (32 %) or parent (36 %), while a smaller proportion provides care to relatives (18 %) or friends (18 %) (Colombo et al. 2011: 90). There is a question as to how far the number of informal caregivers will increase in the years ahead in response to the cutbacks in publicly funded care. This question is even more pertinent in the light of other societal developments, such as shrinking family size, the increased labour market participation of women and the rising retirement age, which may limit the supply of informal caregivers in the near future (Agree and Glaser 2009; Sadiraj et al. 2009).

As the prevalence of informal care at population level reflects how many persons do take up the caregiver role, a theoretical answer to this question starts at the individual level. We chose a theoretical perspective in which individuals are assumed to weigh pros and cons to take up a caregiving role in a specific social relationship with a sick or vulnerable person, and in which the intention to provide care is facilitated by their embeddedness in the social context, being the family, the social network, and the community. More insight in how macro-level developments impact the individual’s disposition and contextual opportunities for informal caregiving then helps us understand whether and why the volume of informal care will increase in the near future. This paper has two objectives: (1) to provide a basic theoretical framework—the Informal Care Model—that defines which mechanism at the individual, relational, family, network and community level drives informal care provision; and (2) to discuss how current policy and societal developments may impact on mechanisms underlying the provision of informal care.

## The informal care model

**Fig. 1:**
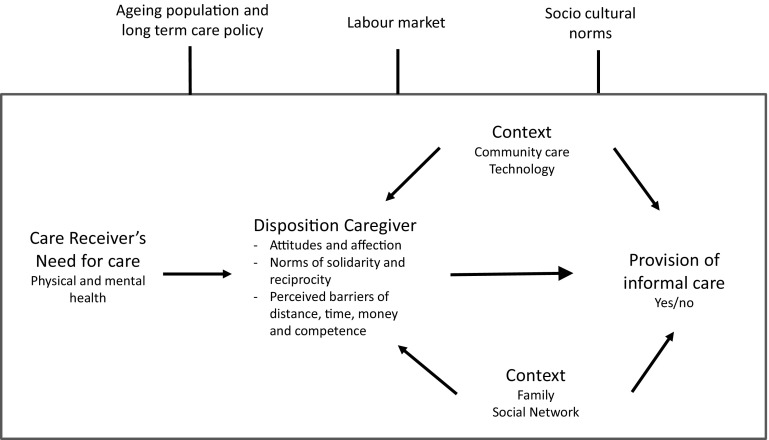
The Informal Care Model: determinants of informal care provision at the individual level

Theoretical notions in the domain of informal care generally focus on *outcomes* of caregiving such as caregiver burden, wellbeing and health (e.g. Pearlin et al. 1990), but less on socio-psychological processes and the societal context that influence the *provision* of informal care. We present the Informal Care Model (ICM), a behavioural model focusing on the individual caregiver that entails three central propositions. First, informal caregiving starts with the notion that someone in the social network is in need of care. Second, individual dispositional factors predict to what degree one *intends* to provide care. Third, whether one will actually *provide* care depends on external conditions that facilitate or restrict the provision of care. As such, the provision of informal care is depicted as a process in which individual, relational and contextual factors of both care recipient and caregiver are intertwined. Although the care process in real life is dynamic in nature and involves multiple actors (care recipient, caregivers, professionals), we limit the description of the ICM to its basic elements and describe it from the perspective of the informal caregiver only. In explicating which dispositional factors and external conditions of the caregiver are at stake here, we will use a general behavioural model, the Theory of Planned Behavior (Fishbein and Azjen 2010), and theoretical frameworks in the care and support domain, viz. the Behavioral Model of Health Service Use (Andersen and Newman 2005) and the Intergenerational Solidarity Framework (Bengtson and Roberts 1991). The ICM is depicted in Fig. 1.

## The outcome: the onset of informal care provision

Informal care may vary in intensity, type of help provided, location and duration of care provided. In the ICM described below, we disregard the difference between the onset of caregiving and continuity of caregiving, as well as the type of care that is provided. We also limit our theoretical framework to informal caregivers of community-dwelling care recipients, as informal caregiving in residential settings presents other choice options and will not be discussed here. The focus is on the arguments that predict to what degree people are likely to start providing care.

## Starting point: the need to provide care

The Behavioral Model of Health Service Use (Andersen and Newman 2005) states that the need for care is the first trigger for use of care. Empirical research has consistently shown that the health status of the care recipient, as indicator for need of care, is the most important determinant for use of care (Babitsch et al. 2012). Also, in theoretical frameworks that focus on the outcomes of informal caregiving, for example the stress-coping model of Pearlin et al. (1990), the health status of the care recipient is regarded as the ‘primary objective stressor’, which directly elicits the provision of informal care. In the ICM, the physical and mental health status of the care recipient is thus an important driver for the onset of informal caregiving.

## Individual disposition of caregivers

In line with the Theory of Reasoned Action (Fishbein and Azjen 2010), we argue that the intention of a specific behaviour, in this case informal care provision, is based on general beliefs (is this what I want to do?), normative beliefs (is this what I have to do?) and perceived constraints (is this what I can do?). As informal care is always provided in the relationship between caregiver and receiver, these dispositional factors are in part relationship-specific.

### Do I want to? Attitudes and affection

Caregiving is in part driven by motives and values that are rooted in socialisation, educational experiences and family backgrounds (Burr et al. 2005). A strong general concern for helping others may thus be driven by feelings of societal responsibility (altruism; Burr et al. 2005), religious beliefs and involvement (Goodman et al. 1997) or gender-related role expectations of women as caregivers (Miller 1990). Another example of general care attitudes is how strongly one adheres to the general norm that family (rather than the government) is responsible for helping others in times of need (Cooney and Dykstra 2011). Hochschild (1995) speaks in this respect of ‘moral framing rules’, meaning that if a person feels that there is sufficient support provided by the government, he or she has a lower intention to provide (very intense) informal care. A large majority of the population in Western European countries favours government responsibility in this respect, especially in the Netherlands and the Scandinavian countries (Haberkern and Szydlik 2010; Suanet et al. 2012), but individual variation within countries also exists. Those who prefer informal care to formal care have been shown to be more likely to provide informal care than those who prefer formal care to informal care (Pinquart and Sörensen 2002).

In addition to general care dispositions, we also specify relationship-specific motivations. A useful framework here is the Intergenerational Solidarity Framework (Bengtson and Roberts 1991), which posits six dimensions defining the degree to which a child is likely to support a parent (or vice versa). Two of those, affectional and associational solidarity, both indicate the strength of the personal bond or the quality of the relationship with the care recipient; the stronger the bond, indicated by high levels of affection and frequent contact, the greater the likelihood to provide care (Silverstein et al. 1995, 2008).

### Do I have to? Normative beliefs

Norms of reciprocity and norms of solidarity are driving forces, in particular at the relational level (Bengtson and Roberts 1991). Reciprocal solidarity refers to the wish to keep a balanced exchange of support in the relationship. According to this line of reasoning, informal care is provided because the care recipient has invested considerably in the relationship in the past and ‘deserves’ a return on those investments. Normative solidarity indicates the degree to which someone feels ‘obliged’ or ‘expected’ to provide care, something that may have both positive and negative connotations. Consensus on norms of solidarity between care receiver and caregiver is another dimension in the framework of intergenerational solidarity. It refers to the degree that care recipient and caregiver agree on what is expected in terms of informal caregiving. Empirical studies focus in this respect on parent–child relationships and show that children may be ambivalent about providing care despite the parents’ expectations being high (Pillemer and Suitor 2006). A greater consensus between care receiver and caregiver regarding strong norms of reciprocity and solidarity will increase the likelihood of (intense) informal care provision.

### Can I? Perceived barriers to provide care

#### Distance

Having to cover a geographical distance in order to provide in-home care or help with transportation may be an important barrier to caregiving (Smits et al. 2010) to both children (Silverstein, Conroy and Gans 2008) and non-kin (Barker 2002; Lapierre and Keating 2013; Egging et al. 2011). For some adult children, the single status of the care recipient may even be the trigger for co-residency (Seltzer and Friedman 2014).

#### Time

The caregiver may perceive opportunity costs that limit the freedom to provide care. There are time constraints, resulting from having a paid job, being engaged in volunteering or looking after a family with young children (see the review by Bauer and Sousa-Poza 2015). Paid employment limits taking up the caregiver role, because people with long working hours less often start providing care than people who work shorter hours and non-workers (Josten and De Boer 2015). Regarding marital status, singles have more time to provide care (Pezzin and Schone 1999), but married caregivers are often assisted by their spouses in family chores, which enables them to maintain their caregiver and other roles in life. These effects may compensate for each other, which may explain why marital status and family roles have relatively little impact on the provision of caregiving after correcting for other correlates (need for care, geographical distance to care recipient) (Dwyer and Coward 1991; Silverstein et al. 2008).

#### Money

A perceived barrier to provide informal care may be the financial costs involved in travelling towards the care recipient, taking up leave from work, or costs involved in buying gifts or goodies for the care recipient. There is also evidence that caregivers have a lower employment rate than persons who never taken on that role and that they are more likely to work in unskilled (non-career track) occupations (Carmichael et al. 2010). Still, only a small part of the informal caregivers experience financial problems due to the caregiving (Hoefman et al. 2011). On the other hand, money may be a trigger to provide care, in particular, if one receives benefits for informal care provision, which is the case in long-term care systems with cash-for-care benefits (Hammer and Österle 2003).

#### Competence

Another type of perceived costs concerns the physical and mental health capacities of the caregiver that define whether one feels up to provide the care needed by the care recipients. A review study showed that poor health generally limits the provision of care (Bauer and Sousa-Poza 2015). Competence is enlarged if one has the knowledge and skills to provide care, which explains why those who work in the care sector are overrepresented among informal carers. This category of employees is also more familiar with locating relevant public bodies for care recipients, which leads them to take up the caregiving role more often (Kooiker and de Boer 2008).

## Context

**Fig. 2:**
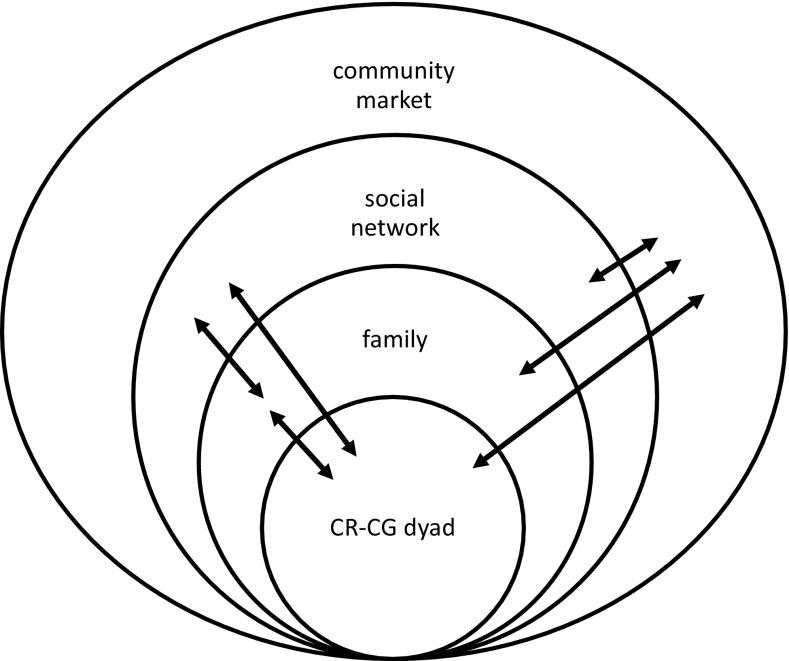
Interdependence in caregiving between four levels of caregivers

Whether the individual intention to provide informal care results in the actual provision of care is facilitated or restricted by contextual factors, in particular, the presence of other types of helpers in family, the larger social network and the community. On the individual level, the presence of other (potential) caregivers may impact on caregiving via individual dispositions (e.g. normative pressure) or via the opportunity structure of the care network (e.g. community care or technology), as pictured in Fig. 1. Depending on the context, the presence of other helpers may have a positive effect (indicating complementarity) or a negative effect (indicating substitution) on caregiving. On a network level, both structural and normative aspects of the social context may underlie substitution and complementarity within one level (e.g. the family), but also between two or more levels (e.g. family versus community). The interdependence between different levels of caregivers is illustrated in Fig. 2.

### Family

Within families, the spouse is the preferred informal caregiver and, since he/she lives in the same household, is generally seen as the most suitable person for the task (Litwak 1985; Cantor 1979). Where a spouse is present, it is thus very likely that he/she provides care, alone or with a little help from one or two children or other helpers (Jacobs et al. 2016). Where no spouse is present or the spouse has limited capacity to provide care due to health problems of their own, children are more likely to provide care than other relatives or non-kin. For spouses, therefore, the likelihood of providing care less often depends on the help received from children, whereas for children, the absence of a spouse is an important trigger for providing care (Jacobs et al. 2016).

Many studies focus on the caregiver selection among children within families. The size and composition of the family seems to be crucial in this respect (e.g. Stuifbergen et al. 2008). Recent studies have shown that siblings positively affect each other’s decision to provide care, as they seem to decide mutually that sharing the care has advantages for everybody (Tolkacheva et al. 2011). Who is providing care among the siblings also depends on the individual characteristics and resources of the siblings. Those who live closer than others, have a better bond with their parents or have more time available, are more likely to provide care (Tolkacheva et al. 2010; Szinvovacz and Davey 2013; Silverstein et al. 2008). In addition to characteristics of the children, parental filial expectations contribute to determining which of the children were more likely to provide care in times of need (Pillemer and Suitor 2013). This suggests that consensual solidarity between parents and children, in addition to solidarity among siblings, is an important family-level determinant in individual informal caregiving.

### The social network

The interdependence of choices can be extended beyond the family and include other members of the social network as well, as evidenced by the composition of the care networks of older adults (Keating et al. 2003; Broese van Groenou et al. 2015). Other relatives, neighbours and other non-kin often act as assistants to the core informal caregivers, i.e. the spouse or children (Cantor 1979). As such, their caregiving is likely to be complementary to that of spouses and children (Lapierre and Keating 2013; Egging et al. 2011). Recent studies on the composition of care networks of older adults show that spouses share almost none of the care with other informal caregivers, whereas children are likely to collaborate with relatives and other non-kin (Jacobs et al. 2016; Fast et al. 2004). This suggests that the presence of other network members may not impact on spousal care provision, whereas a stronger positive association is to be expected for child and other types of caregivers.

### Care in the community

Many empirical studies report a negative association between the use of formal and informal care (Li 2005; Swinkels et al. 2015; Geerts and Van den Bosch 2012), which corroborates the notion that a lack of formal care, such as professional (publicly funded) home carers, privately paid caregivers and volunteers, facilitates the use of informal care and vice versa. The use of community services such as assistive devices, meals-on-wheels, paid transportation and support services for informal caregivers may also substitute for the care provided by informal caregivers, especially as regards personal care and nursing care (Li 2004). Regarding the onset of informal caregiving, there may thus be a negative association with the use of community care and services. However, in the long run with increased need for care, there is evidence that a complementary or supplementary model is more likely than a substitution model (e.g. Peek et al. 1997; Allen et al. 2012).

## Applicability of the ICM

The ICM was designed to study the onset of informal caregiving in a general population and may be applied in empirical studies to explain individual variety in informal care provision, e.g. by gender, socio-economic status, and type of relationship. But it may also be applied to answer more complex research questions involving longitudinal, multi-actor or cross-national designs. The changes in the intensity of informal caregiving, for example, may be explained from an increased need for care, but also from a deceased lack of competence due to caregiver burden or poor health. This requires that outcomes of caregiving are also included in the model and hypotheses are developed on causality in the process of caregiving. Using the model to explain cross-national differences in informal care giving requires a detailed outline of the structures of provision such as the allocation of publicly paid care and support services, as this may define the positive or negative association between informal care and formal care. Applicability of the ICM in empirical studies thus asks for further specification of the determinants, the outcome variable, and/or of the effects of these determinants on the outcome variable in use.

## Macro-level structures of provision

A second aim of this paper is to discuss the degree to which policy and societal changes may impact on the mechanisms that underlie informal care provision and that were outlined above. Although there are many societal changes to consider with regard to informal caregiving (Agree and Glaser 2009), we will focus on three domains in which illustrative changes may affect the need of care recipients, disposition of (potential) caregivers and contextual factors for the provision of care: long-term care and population ageing; the labour market; and the socio-cultural context. We will discuss the degree to which changes in these domains may positively or negatively affect the likelihood of informal care provision to older or dependent people. Examples of the arguments are given in Table 1.

**Table 1:** Examples of how changes in long-term policy, the labour market and the socio-cultural domain may impact on the determinants of providing informal care

	Long-term care policy and population ageing	Labour market	Socio-cultural
Care recipient’s need for care	Cutbacks in professional home care and residential care and an increase of the oldest old in the population—> *increased need* for long-term complex care at home		
Caregiver’s disposition to provide care	Public discourse on civic norms and responsibilities and call for ‘norm of solidarity’—>*increased* general disposition to provide help to others among kin and non-kin	Women reconsider the value of a career after middle age compared to the value of caring for older parents—> *decreased* intention to provide care Raised retirement age—> *reduced* time and health capacity for informal care provision as perceived by older employees	The norm of reciprocity transforms into the norm of solidarity among non-kin—>*increased* care provided by friends and neighbours Weakened norm of solidarity in modern families—> *reduction in* care provided in step-families and second marriages and LAT-relationships
Context	Availability of community services, support services for informal caregivers, technological devices—> *delays*, *complements* and *facilitates* informal care	Support provided at the workplace to continue working whilst caring—> *facilitates* informal care	More helpers among kin and non-kin resulting in *larger* care networks—> *complements* and facilitates informal care provided by partner and child

### Long-term care and population ageing

The reform of long-term care entails a reduction in the intensity of professional care provided at home and more strict criteria for admission to residential care, both resulting in an increased *need for care* among community-dwelling older adults. Given the projected doubling of the population aged over 80 over the coming decades (Colombo et al. 2011), many more people will stay at home and need personal care, nursing care and other types of care. In particular, those with complex cognitive and/or physical impairments, such as Alzheimer’s disease, or at end of life, will need intensive and long-term home care. As the need for care is the most important trigger to start caregiving, the ICM predicts that more people in need of complex care, due to the ageing of the population and the process of further de-institutionalising, will contribute to more people providing informal care.

The current normative discourse on informal care (Da Roit 2013) aims to weaken the reliance on government responsibility as care provider and to strengthen the norm of providing care to close relatives as well as to non-kin. In our conceptual model, this argument is reflected in the caregiver’s general disposition to provide care. Cross-national comparisons show that in countries with stronger family norms, individuals are more likely to provide informal care (Haberkern and Szydlik 2010; Cooney and Dykstra 2011). A recent study in the Netherlands showed that there has already been a slight shift in recent years towards agreeing that the family carries more responsibility (rather than the government) for providing care in old age (Verbakel 2014). A shift in family-state responsibility is more likely to change the nature of informal care (in hours or types of tasks) and not the decision to care per se, but it is also known that feelings of being needed and obligation increase the likelihood of informal care provision (Oudijk et al. 2011). If the current normative discourse shifts the public view towards more family responsibility and thus strengthens the feelings of being needed, this may contribute to more people taking up the caregiver role. Although the discourse is mainly focused on family responsibilities, it may affect the feeling of being needed among non-kin as well and lead more non-kin to take up a caregiver role, albeit in lower intensity and in different types of tasks than (close) kin caregivers generally hold. This normative discourse may thus contribute to care networks that are larger in size and more diverse in composition than before with close kin as primary caregivers and other relatives and non-kin as assistive secondary caregivers.

The reform of long-term care is accompanied (in the Netherlands) by a decentralisation of the allocation of home care from national to community level. Only residential care is in our country still governed at the national level. This puts local authorities in charge of the local organisation of care teams which combine health and social care with professional household, personal and nursing care. These care teams aim for more self-management among older care recipients and a larger reliance on their social network, but also work towards facilitating informal caregiving by providing more support services for informal caregivers (Lamura et al. 2008). As families may live at a greater distance, the general aim is to increase support from local non-kin, neighbours, friends and volunteers. Civic neighbourhood organisations also become an important local player in arranging community care, adding to the potential rise of the number of non-kin caregivers. Also, in the technological domain, many digital tools and technical devices (e.g. GPS sensors) are being developed in order to allow care recipients to remain at home for as long as possible and potentially reduce the burden of caregivers (Agree et al. 2005). Some of these services and tools can be purchased on the market, others are paid for partly by the local authority. This results in a wide range of services and devices that may delay the onset of informal care, and facilitate the provision of informal care in the long run.

### Labour market

There are several developments on the labour market that may affect the determinants of informal care provision. First, population ageing has led to the raising of the retirement age in many European countries. In the Netherlands, the retirement age will be raised from 65 to 67 years in 2021. This directly affects the time and income available for informal care provision in this specific age group, but it may also affect potential caregivers below the age of 65. According to the literature, participation on the labour market competes with informal care provision, at least for those who work full-time (Josten and De Boer 2015). It can be argued that an increased need for informal care may lead people to reconsider possible career moves; women, in particular, might not take up full-time positions after the age of 45–55, as they may also face the prospect of providing care to their parents(-in-law). In the Netherlands, women’s organisations genuinely fear that the increased need for informal care could reverse the trend of women’s increased participation on the labour market (NVR 2014). This may in particular be the case for migrant women and those with lower socio-economic status, traditionally groups that are involved in caregiving. On the other hand, employers are increasingly aware that their (older) employees face the need to juggle roles in work and informal care, and that this requires a change of attitude and formal arrangements within their organisation. Many studies have shown that organisational support reduces caregivers’ stress at work (Plaisier et al. 2015) and stops informal caregivers (especially women) from giving up their jobs (Pavalko and Henderson 2006). In sum, perceived barriers to caregiving are both positively and negatively affected by the developments on the labour market, in particular for close kin.

### Socio-cultural domain

Socio-cultural norms may differ between individuals (see ‘Individual disposition of caregivers’) but are also expressed at the macro-level within countries. Two societal developments may affect both the structure of and norms within the care networks of those in need of care. The first concerns a possible shift in norms of solidarity within the family and the social network. Due to processes of individualisation the distinction between kin and non-kin has become more blurred in recent decades (Allan 2008). Family relations have become more volatile and individuals are less dependent solely on their family for emotional, social and economic support. Friendships, on the other hand, have gained importance and become more prominent in the personal networks of older adults in recent decades (Suanet et al. 2013). For some persons, friends are ‘doing family’ including taking up the caregiver role (Allan 2008). They describe social relations that are not based on blood or marriage ties as ‘fictive kinship’; these relationships might provide informal support as family substitutes. This implies that friends may also play a more prominent role in the informal care network in times of need. Second, family structures have taken on the vertical shape of a ‘beanpole’, due to lower fertility and longer lives of the oldest generations (Bengtson et al. 1990), thus reducing the number of potential caregivers in the family (Ryan et al. 2012). Family structure has also become more complex in recent decades due to increased rates of divorce and remarriage (Pezzin et al. 2008). Relationships with stepchildren or with partners that develop later in life may lack the quality and strong normative solidarity that is so important for informal care provision. Where the increased relevance of non-kin in the personal network may increase the likelihood of informal care provision by non-kin, therefore, new types of kin relationships may inhibit the provision of informal care in times of need.

## Conclusion

We developed the Informal Care Model (ICM) in order to describe the multiple and diverse arguments of an individual to take up the caregiver role when confronted with someone in need of care. In recent decades, separate strands of literature have focused on the outcomes of caregiving (such as caregiver burden, positive evaluations and well being), on the association between work and care, on intergenerational solidarity, or on the links between formal and informal care. Our conceptual model aimed to include many of these aspects, as this enhances our understanding of why individual informal caregivers (spouse, child, relative, non-kin) differ in taking up the caregiver role. The model provides building steps for how informal care provision works out differently in specific contexts, as families, networks and communities, but more theoretical work needs to be done on the processes of substitution and complementarity among these levels of caregivers. We argued that current changes in policy and society may impact both negatively and positively on the mechanisms of the ICM. Given the increased need for care among community-dwelling individuals, the model suggests that more people may take up informal caregiving in the years ahead contributing to large and diverse care networks around the old and dependent. In order to increase the potential of (non-)kin informal carers, we might need additional incentives such as reimbursements, pension benefits, legal obligations, and career benefits. This makes long-term informal care provision a complex phenomenon including multiple actors in various contexts. More research is needed to put the ICM to an empirical test, preferably using information on care recipients, informal caregivers and community care in a dynamic design and in different countries. Such information will increase insight in the developments in informal care provision in retrenching welfare states.

## References

[CR1] Agree EM, Glaser K, Uhlenberg P (2009). Demography of informal caregiving. International handbook of population aging.

[CR2] Agree EM, Freedman VA, Cornman JC, Wolf DA, Marcotte JE (2005). Reconsidering substitution in long term care: when does assistive technology take the place of personal care?. J Gerontol B Soc Sci.

[CR3] Allan G (2008). Flexibility, friendship and family. Pers Rel.

[CR4] Allen SM, Lima JC, Goldscheider FK, Roy J (2012). Primary caregiver characteristics and transitions in community-based care. J Gerontol B Soc Sci.

[CR5] Andersen R, Newman JF (2005). Societal and individual determinants of medical care utilization in the United States. Milbank Q.

[CR6] Babitsch B, Gohl D, von Lengerke T (2012). Re-revisiting Andersen’s behavioral model of health services use: a systematic review of studies from 1998–2011. GMS Psych Soc Med.

[CR7] Barker JC (2002). Neighbors, friends and other nonkin caregivers of community-living dependent elders. J Gerontol Soc Sci.

[CR8] Bauer JM, Sousa-Poza A (2015). Impacts of informal caregiving on caregiver employment, health and family. Pop Ageing.

[CR9] Bengtson VL, Roberts REL (1991). Intergenerational solidarity in aging families: an example of formal theory construction. J Marriage Fam.

[CR10] Bengtson VL, Rosenthal CJ, Burton LM, Binstock RH, George LK (1990). Paradoxes of families and aging. Handbook of aging and the social sciences.

[CR11] Broese van Groenou M, Jacobs M, Zwart-Olde I, Deeg DJH (2015). Mixed care networks of community-dwelling older adults with physical health impairments in the Netherlands. Health Soc Care Community.

[CR12] Burr JA, Choi NG, Mutchler JE, Caro FG (2005). Caregiving and volunteering: are private and public helping behaviors linked?. J Gerontol Soc Sci.

[CR13] Cantor MH (1979). Neighbors and friends: an overlooked resource in the informal support system. Res Aging.

[CR14] Carmichael F, Charles S, Hulme C (2010). Who will care? Employment participation and willingness to supply informal care. J Health Econ.

[CR15] Colombo F et al (2011) Help Wanted? Providing and paying for long-term care. OECD Health Policy Studies, OECD Publishing. http://www.oecd.org/health/longtermcare/helpwanted

[CR16] Cooney TM, Dykstra PA (2011). Family obligations and support behavior: a United States–Netherlands comparison. Ageing Soc.

[CR17] Da Roit B, Ranci C, Pavolini E (2013). Long-term care reforms in the Netherlands. Reforms in long-term care policies in Europe.

[CR18] Dwyer JW, Coward RT (1991). A multivariate comparison of the involvement of adult sons versus daughters in the care of impaired parents. J Gerontol.

[CR19] Egging S, De Boer AH, Stevens NL (2011). Zorgzame vrienden en buren als mantelzorgers van oudere volwassenen: een vergelijking met kinderen [Caring friends and neighbors as a predictor of caregiver strain]. Tijdschr Gerontol Geriatr.

[CR20] Fast J, Keating NC, Derksen L, Otfinowski P (2004). Characteristics of family/friend care networks of frail seniors. Can J Aging.

[CR21] Fishbein M, Azjen I (2010). Predicting and changing behavior: the reasoned action approach.

[CR22] Geerts J, Van den Bosch K (2012). Transitions in formal and informal care utilisation among older Europeans: the impact of national contexts. Eur J Ageing.

[CR23] Goodman CR, Zarit SH, Steiner VL (1997). Personal orientation as a predictor of caregiver strain. Aging Ment Health.

[CR24] Haberkern K, Szydlik M (2010). State care provision, societal opinion and children’s care of older parents in 11 European countries. Ageing Soc.

[CR25] Hammer E, Österle A (2003). Welfare state policy and informal long-term care giving in Austria: old gender divisions and new stratification processes among women. J Soc Policy.

[CR26] Hochschild AR (1995). The culture of politics: traditional, postmodern, cold-modern, and warm-modern ideals of care. Soc Politics.

[CR27] Hoefman RJ, van Exel NJ, de Jong SL, Redekop WK, Brouwer WB (2011). A new test of the construct validity of the CarerQoL instrument: measuring the impact of informal caregiving. Qual Life Res.

[CR28] Jacobs MT, Aartsen M, Deeg DJH, Broese van Groenou M (2016). Diversity in older adults’ care networks: the added value of psychological factors and social network proximity. J Geront B Psychol Sci Soc Sci.

[CR29] Josten E, De Boer A (2015) Concurrentie tussen betaald werk en mantelzorg [Competition between paid work and informal care]. The National Institute for Social Research, The Hague, the Netherlands

[CR30] Keating N, Otfinowski P, Wenger C, Fast J, Derksen L (2003). Understanding the caring capacity of informal networks of frail seniors: a case for care networks. Ageing Soc.

[CR31] Kooiker S and de Boer A (2008). Portretten van mantelzorgers [Portraits of informal caregivers]. The National Institute for Social Research, the Hague, the Netherlands

[CR32] Lamura G (2008). Family Carers’ Experiences Using Support Services in Europe: empirical Evidence From the EUROFAMCARE Study. Gerontol.

[CR33] Lapierre TA, Keating N (2013). Characteristics and contributions of non-kin carers of older people: a closer look at friends and neighbours. Ageing Soc.

[CR34] Li LW (2004). Caregiving network compositions and use of supportive services by community-dwelling dependent elders. J Gerontol Soc Work.

[CR35] Li LW (2005). Longitudinal changes in the amount of informal care among publicly paid home care recipients. Gerontol.

[CR36] Litwak E (1985). Helping the Elderly: The complementary roles of informal networks and formal systems.

[CR37] Miller B (1990). Gender differences in spouse caregiver strain: socialization and role explanations. J Marriage Fam.

[CR38] NVR (2014). Brief aan de ministers van SZW en OCW en de staatssecretaris van VWS: decentralisatie van beleid en de gevolgen voor vrouwen [Letter to governmental departments on decentralization and the consequences for women]. Nederlandse Vrouwen Raad [National Women’s Council], the Hague, the Netherlands

[CR39] Oudijk D, Woittiez I, De Boer A (2011). More family responsibility, more informal care? The effects of motivation on the giving of informal care by people aged 50 or over in the Netherlands compared to other European countries. Health Policy.

[CR40] Pavalko EK, Henderson KA (2006). Combining care work and paid work: do workplace policies make a difference?. Res Aging.

[CR41] Pavolini E, Ranci C (2008). Restructuring the welfare state: reforms in long-term care in Western European countries. J Eur Soc Policy.

[CR42] Pearlin LI, Mullan JT, Semple SJ, Skaff MM (1990). Caregiving and the stress process: an overview of concepts and their measures. Gerontology.

[CR66] Peek CW, Zsembik BA, Coward RT (1997). The changing caregiving networks of older adults. Res Aging.

[CR43] Pezzin LE, Schone BS (1999). Parental marital disruption and intergenerational transfers: an analysis of lone elderly parents and their children. Demography.

[CR44] Pezzin LE, Pollak RA, Steinberg Schone B (2008). Parental marital disruption, family type, and transfers to disabled elderly parents. J Gerontol Soc Sci.

[CR45] Pillemer K, Suitor JJ (2006). Making choices: a within-family study of caregiver selection. Gerontol.

[CR46] Pillemer K, Suitor JJ (2013). Who provides care? A prospective study of caregiving among adult siblings. Gerontol.

[CR47] Pinquart M, Sörensen S (2002). Older adults’ preferences for informal, formal, and mixed support for future care needs: a comparison of Germany and the United States. Int J Aging Hum Dev.

[CR48] Plaisier I, Broese van Groenou M, Keuzenkamp S (2015). Combining work and informal care: the importance of caring organisations. Hum Res Manag J.

[CR49] Ryan LH, Smith J, Antonucci TC, Jackson JS (2012). Cohort differences in the availability of informal caregivers: are the Boomers at risk?. Gerontol.

[CR50] Sadiraj K, Timmermans J, Ras M, De Boer A (2009) De toekomst van de mantelzorg [The future of informal care]. The National Institute for Social Research, the Hague, the Netherlands

[CR51] Schulz R, Mittelmark M, Burton L, Hirsch C, Jackson S (1997). Health effects of caregiving: an ancillary study of the cardio vascular health study. Ann Beh Med.

[CR52] Seltzer J, Friedman EM (2014). Widowed mothers’ co-residence with adult children. J Gerontol Soc Sci.

[CR53] Silverstein M, Parrott TM, Bengtson VL (1995). Factors that predispose middle-aged sons and daughters to provide social support to their older parents. J Marriage Fam.

[CR54] Silverstein M, Conroy SJ, Gans D, Szinovacz ME, Davey A (2008). Commitment to caring: filial responsibility and the allocation of support by adult children to older mothers. Caregiving contexts: cultural, familial, and societal implications.

[CR55] Smits A, van Gaalen R, Mulder CH (2010). Parent-child co-residence: who moves in with whom and for whose needs?. J Marriage Fam.

[CR56] Stuifbergen MC, van Delden JJM, Dykstra PA (2008). The implications of today’s family structures for support giving to older parents. Ageing Soc.

[CR57] Suanet B, Broese van Groenou M, Van Tilburg T (2012). Informal and formal home-care use among older adults in Europe: can cross-national differences be explained by societal context and composition?. Ageing Soc.

[CR58] Suanet B, Van Tilburg TG, Broese van Groenou MI (2013). Nonkin in older adults’ personal networks: more important among later cohorts?. J Gerontol Soc Sci.

[CR59] Swinkels JC, Suanet B, Deeg DJH, Broese van Groenou M (2015). Trends in the informal and formal home care use of older adults in the Netherlands between 1992 and 2012. Ageing Soc.

[CR60] Szinvovacz ME, Davey A (2013). Prevalence and predictors of change in adult-child primary caregivers. Int J Aging Hum Dev.

[CR61] Tolkacheva N, Broese van Groenou MI, Van Tilburg TG (2010). Sibling Influence on care given by children to older parents. Res Aging.

[CR62] Tolkacheva T, Broese van Groenou MI, De Boer A, Van Tilburg T (2011). The impact of informal care-giving networks on adult children’s care-giver burden. Ageing Soc.

[CR63] Triantafillou J et al (2010) Informal care in the long-term care system. European Overview paper. Athens/Vienna: Interlinks. http://interlinks.euro.centre.org

[CR64] Verbakel E (2014) Toenemende publieke steun voor meer eigen verantwoordelijkheid in de zorg? [Increasing public support for more responsibility in care?] Bestuurswetenschappen 68:3–23

[CR65] Verbeek-Oudijk D, Woittiez I, Eggink E, Putman L (2014). Who cares in Europe? A comparison of long-term care for the over-50 s in sixteen European countries.

